# Efficacy and safety of fruquintinib combined with albumin‐bound paclitaxel as second‐line therapy for advanced gastric cancer following failure of PD‐1 inhibitor‐containing treatment (TACTIC GC‐01): A phase II single‐arm study

**DOI:** 10.1002/ijc.70299

**Published:** 2025-12-25

**Authors:** Xiaoting Ma, Kai Ou, Xiu Liu, Biyang Cao, Wenwei Yang, Jingyu Lu, Letian Zhang, Qi Wang, Lizhen Gao, Zhichao Jiang, Yongkun Sun, Lin Yang

**Affiliations:** ^1^ Department of Medical Oncology National Cancer Center/National Clinical Research Center for Cancer/Cancer Hospital, Chinese Academy of Medical Sciences and Peking Union Medical College Beijing China; ^2^ Department of Internal Medicine Peking Union Medical College Hospital, Chinese Academy of Medical Sciences and Peking Union Medical College Beijing China; ^3^ Department of Medical Oncology Beijing Chaoyang Sanhuan Cancer Hospital Beijing China; ^4^ Department of Medical Oncology Beijing Chaoyang Huanxing Cancer Hospital Beijing China

**Keywords:** albumin‐bound paclitaxel, fruquintinib, gastric adenocarcinoma, PD‐1, second‐line

## Abstract

This study (TACTIC GC‐01) aimed to evaluate the efficacy and safety of fruquintinib combined with albumin‐bound paclitaxel as a second‐line treatment for advanced gastric cancer (GC) following progression on programmed cell death protein 1 (PD‐1) inhibitor‐based therapy. In this single‐center, single‐arm, prospective trial, patients with metastatic gastric adenocarcinoma who failed first‐line anti‐PD‐1 combined with chemotherapy treatment received six cycles of albumin‐bound paclitaxel combined with fruquintinib, followed by fruquintinib maintenance monotherapy. The primary endpoint was progression‐free survival (PFS), while secondary endpoints included overall survival (OS), objective response rate (ORR), disease control rate (DCR), and adverse event (AE) incidence. Between February 24, 2022, and March 26, 2024, 41 patients were enrolled, with three receiving only one treatment cycle. The safety analysis included all 41 patients, while the full analysis set comprised 38 patients. Median PFS and OS were 5 months and 14 months, respectively, with corresponding 6‐ and 12‐month PFS rates of 31.7% and 17.3%, and OS rates of 87.8% and 51.7%, respectively. Log‐rank analysis identified frontline immunotherapy duration (≥3 cycles) as a key risk factor for PFS, while metastasis to ≥2 organs significantly impacted OS. The ORR and DCR were 44.7% and 94.7%, respectively. Treatment‐related AEs occurred in 90.2% of patients, with grade 3–4 AEs (notably neutropenia and thrombocytopenia) observed in 41.5% of them. These findings suggest that fruquintinib plus albumin‐bound paclitaxel exhibits promising efficacy and manageable toxicity in anti‐PD‐1‐refractory GC, warranting further exploration in combination strategies targeting alternative pathways.

## Graphical abstract

What's new?

Anti‐angiogenic drugs have shown promising efficacy as a second‐line treatment for advanced gastric cancer. However, it remains unclear how alterations in the tumor microenvironment following first‐line immunotherapy may impact tumor angiogenesis and influence subsequent therapeutic outcomes. This single‐arm study prospectively explored the efficacy and safety of the anti‐angiogenic drug fruquintinib combined with albumin‐bound paclitaxel as a second‐line treatment for gastric cancer after immunotherapy combined with chemotherapy. The results suggest that chemotherapy combined with anti‐angiogenic drugs can achieve good efficacy and manageable toxicity in an immunotherapy‐exposed population, potentially enriching the second‐line treatment landscape for patients with gastric cancer.

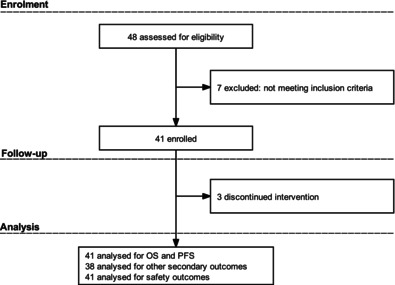

**Abbreviations**
AE: adverse event
CI: confidence interval
CR: complete response
DCR: disease control rate
ECOG: Eastern Cooperative Oncology Group
FAS: full analysis set
GC: gastric cancer
HER2: human epidermal growth factor receptor 2
HR: hazard ratio
ICI: immune checkpoint inhibitor
mDoR: median duration of response
ORR: objective response rate
OS: overall survival
PD: progressive disease
PD‐1: programmed cell death protein 1
PFS: progression‐free survival
pMMR: proficient mismatch repair
PR: partial response
PS: Performance Status
SD: stable disease
SSS: safety analysis set
VEGFR: vascular endothelial growth factor receptor

## 1 INTRODUCTION

Gastric cancer (GC) remains a leading global health challenge as one of the most prevalent malignant tumors. Based on the latest GLOBOCAN 2022 data, this disease accounts for approximately 970,000 new cases annually worldwide and 660,000 related deaths, ranking fifth in incidence and mortality rates among all cancers.^1^ The poor prognosis for advanced cases is particularly concerning, where the five‐year survival rate typically falls below 20%, highlighting the urgent need for improved diagnostic and therapeutic strategies.^2^

Currently, novel therapies, such as immunotherapy and anti‐Claudin18.2 agents, have been incorporated into the first‐line treatment for GC, providing new hope for certain patients. However, progress for patients receiving second‐line therapy remains limited. Anti‐angiogenic therapy targeting vascular endothelial growth factor receptor (VEGFR) is a key strategy in the treatment of GC and serves as a viable option after the failure of standard therapy. Based on the RAINBOW‐Asia trial, ramucirumab was approved in 2022 as a second‐line treatment for GC. While the combination of ramucirumab and paclitaxel improves progression‐free survival (PFS), it does not significantly enhance overall survival (OS).^3^ Consequently, treatment options for patients with advanced GC who experience progression after first‐line therapy remain scarce, underscoring a critical unmet clinical need for more effective therapeutic approaches.

Fruquintinib is a small‐molecule quinazoline inhibitor of angiogenesis that targets the VEGFR kinase family, including VEGFR‐1, VEGFR‐2, and VEGFR‐3. This molecule inhibits the phosphorylation of VEGFR on the surface of vascular endothelial cells and obstructs its downstream signaling pathways, thereby reducing the proliferation, migration, and tube formation of vascular endothelial cells. Ultimately, this leads to the suppression of tumor neovascularization and exerts an anti‐tumor effect.^4^ The FRUTIGA study is a phase III clinical trial that evaluated the efficacy of fruquintinib in combination with paclitaxel as a second‐line treatment for gastric and gastroesophageal junction adenocarcinoma.^5^ A total of 703 patients were enrolled in the study, with 699 of them receiving the treatment. Compared to paclitaxel alone, the combination of fruquintinib and paclitaxel significantly prolonged median PFS (5.6 months vs. 2.7 months, *p* <.0001) and median duration of response (5.5 months vs. 3.7 months, *p* <.0001). Additionally, the objective response rate (ORR) (42.5% vs. 22.4%, *p* <.0001) and disease control rate (DCR) (77.2% vs. 56.3%, *p* <.0001) were notably higher in the combination group compared to the paclitaxel alone group. Notably, approximately 90% of the participants in the FRUTIGA study had not received prior immunotherapy.

However, given the increasing adoption of immune checkpoint inhibitors (ICIs) as the first‐line treatment of GC,^6^, ^7^, ^8^, ^9^ research is now focused on elucidating how alterations in the tumor microenvironment following ICI treatment may impact tumor angiogenesis and influence subsequent therapeutic outcomes. Therefore, this prospective study aimed to evaluate the efficacy and safety of fruquintinib combined with albumin‐bound paclitaxel as a second‐line therapy for patients with GC who did not respond to prior ICI treatment, aiming to provide evidence‐based guidance for clinical practice.

## 2 PATIENTS AND METHODS

### 2.1 Study design

TACTIC GC‐01 is a single‐center, open‐label, single‐arm phase II clinical trial, which included patients receiving combination therapy consisting of albumin‐bound paclitaxel and fruquintinib. The treatment protocol involved six cycles of this combination therapy. Patients who achieved disease control without progression were subsequently maintained on fruquintinib monotherapy until disease progression, the emergence of unacceptable toxicities, or voluntary withdrawal from the study.

### 2.2 Patients

Eligible patients included those with histologically confirmed metastatic gastric adenocarcinoma who experienced progression after first‐line therapy with ICI and chemotherapy. All patients were human epidermal growth factor receptor 2 (HER2)‐negative and proficient mismatch repair (pMMR). The MMR status was determined by immunohistochemistry. Additional inclusion criteria encompassed: (1) age between 18 and 80 years, (2) Eastern Cooperative Oncology Group (ECOG) Performance Status (PS) <2, (3) the ability to take oral medication, (4) a history of systemic chemotherapy and ICI treatment, (5) an expected survival time of at least 3 months, and (6) evaluable tumor lesions. Additionally, participants were required to have no severe lung, heart, or other significant comorbidities. Adequate organ function was assessed using the following tests, conducted within 14 days prior to enrollment: a white blood cell count between 3.0 and 10.0 × 10^9^ per liter, a neutrophil count ≥1.5 × 10^9^ per liter, and a platelet count ≥100 × 10^9^ per liter. Furthermore, patients were required to have serum aspartate aminotransferase and alanine aminotransferase levels <2.5 times the upper limit of normal, serum total bilirubin levels <1.5 times the upper limit of normal, and serum creatinine levels indicating a creatinine clearance of at least 50 mL/min. Patients were excluded if they met any of the following criteria: poorly managed concurrent illnesses or clinically significant pleural effusion/ascites mandating therapeutic drainage during the 14‐day period preceding study participation. Additionally, individuals with a history of other malignant tumors within the past 5 years, continuous systemic steroid administration, ≥ grade 2 peripheral neuropathy, pregnancy or lactation, or an inability to comply with the planned follow‐up schedule for any reason were excluded from the study.

### 2.3 Sample size calculation

The estimate of PFS as the primary endpoint was based on the following assumptions: (1) a two‐sided alpha of 0.05 and 80% power; (2) assuming that the median PFS of the study population could be extended to 4.8 months given that the median PFS of the historical control was 2.9 months (RAINBOW study); and (3) a follow‐up period of 12 months due to an enrollment rate of 2–3 cases per month. Consequently, the estimated sample size was 37, which increased to 42 after allowing for a dropout rate of 10%.

### 2.4 Therapeutic regimen

Patients received albumin‐bound paclitaxel at a dosage of 100 mg/m^2^ administered on days 1 and 8, and oral fruquintinib at a dosage of 4 mg per day from days 1–14, every 3 weeks. According to the pharmacokinetics, the 4 mg dose maintains an effective drug exposure level while reducing the risk of toxicity. As a down‐dose of the maximum tolerated dose of fruquintinib (5 mg), 4 mg was selected as a better‐tolerated alternative. This combination therapy was intended to continue for six cycles. Patients without evidence of progressive disease (PD) would subsequently receive oral fruquintinib as maintenance therapy, administered as a single agent until disease progression, intolerable toxicity occurred, informed consent was withdrawn, or death ensued. The investigators adhered to institutional guidelines for the premedication of patients with antiemetic agents and growth factors. The drug dosage was modified at the investigator's discretion, with the fruquintinib dose being reduced to a minimum of 3 mg per day during the period from days 1–14.

### 2.5 Assessment

Before the commencement of each treatment cycle, clinical examinations and laboratory assessments were mandatory. These evaluations were rigorously conducted in compliance with the Response Evaluation Criteria in Solid Tumors guidelines (RECIST version 1.1; available at www.cancer.gov) until PD occurred.

The safety evaluation consisted of three key components: documentation of adverse events (AEs), interpretation of laboratory findings, and vital sign measurements. AE assessments were classified according to the National Cancer Institute Common Toxicity Criteria (version 5.0) and graded as follows: grade 1 (mild), grade 2 (moderate), grade 3 (severe or medically significant but not immediately life‐threatening), grade 4 (life‐threatening consequences), and grade 5 (fatal outcome). The causality between all AEs and the study drug was assessed. Additionally, since the frontline treatment in this study protocol involved ICI drugs, the assessment of immune‐related adverse events (irAE) was also performed. A suspected irAE was defined as an event that was considered potentially drug‐related by the investigator and exhibited clinical features (such as diarrhea, rash, hepatitis, pneumonia, etc.) aligning with established immune‐mediated toxicity patterns.

### 2.6 Statistical analysis

The primary endpoint of the study was PFS, while the secondary endpoints included OS, ORR, DCR, and the incidence of AEs. PFS was defined as the duration from the initiation of study treatment to the first documentation of PD (according to RECIST v1.1) or death from any cause, whichever occurred first. OS was defined as the time from the initiation of study treatment to death from any cause. Patients who remained alive and progression‐free were censored at the time of their last disease assessment. ORR was defined as the percentage of patients who achieved a complete response (CR) or partial response (PR). DCR was defined as the percentage of patients with a CR, PR, or stable disease (SD). CTCAE (version 5.0) by the National Cancer Institute served as the standard reference for AE assessment and classification throughout the study. Safety endpoints were based on the safety analysis set (SSS), which included patients who received at least one dose of the protocol treatment and underwent one safety evaluation. In contrast, the full analysis set (FAS), defined as all participants who received ≥1 treatment dose and completed ≥1 tumor evaluation, served as the population for efficacy endpoint analysis. The median follow‐up time for the study cohort was calculated using the reverse Kaplan–Meier method. OS and PFS were estimated using the Kaplan–Meier method, following which survival curves were generated. The log‐rank test was used to compare the survival differences between the groups. Univariate and multivariate analyses were performed using Cox proportional hazards (PH) regression models to estimate hazard ratios (HRs) and their 95% confidence intervals (CIs). The PH assumption was verified using the Supremum test. The point estimates for the ORR and DCR, along with their corresponding 95% CIs, were calculated using the Clopper–Pearson exact method. All analyses employed two‐tailed statistical tests, with statistical significance defined at *p* <.05. Data processing and analyses were performed using R statistical software version 4.0.5 with R Studio interface.

## 3 RESULTS

### 3.1 Patient disposition and characteristics

In total, 48 patients were screened between February 24, 2022, and March 26, 2024, among whom 41 were included in the study group and received a combination of albumin‐bound paclitaxel and fruquintinib (Supplementary Figure 1). The median number of treatment cycles was 5 for albumin‐bound paclitaxel and fruquintinib. The proportion of patients who entered maintenance fruquintinib therapy was 19.5% (8 patients). The dosage of fruquintinib was eventually reduced to 3 mg in seven patients. Three of these patients underwent only a single treatment. Consequently, the SSS and FAS comprised 41 and 38 patients, respectively. Table 1 summarizes the baseline demographic and clinical characteristics of the study population. The cohort showed male predominance (>65%) with a majority (70%) of the patients aged ≤65 years.

**TABLE 1:** Baseline characteristics.

Characteristics	All (*N* = 41)
Age (years)	
Mean (SD)	55.8 (11.88)
Median	59.0
Q1, Q3	47.0, 65.0
Min, max	31, 74
Age group, *n* (%)	
≥65	11 (26.83)
<65	30 (73.17)
Sex, *n* (%)	
Male	27 (65.85)
Female	14 (34.15)
ECOG‐PS, *n* (%)	
0	23 (56.1)
1	18 (43.9)
Lauren type, *n* (%)	
Intestinal type	10 (24.39)
Diffuse type	9 (21.95)
Mixed type	5 (12.20)
Not tested	17 (41.46)
Peritoneal metastasis, *n* (%^a^)	
Yes	12 (29.27)
No	29 (70.73)
Liver metastasis, *n* (%^a^)	
Yes	13 (31.71)
No	28 (68.29)
Number of metastatic organs, *n* (%^a^)	
≥2	20 (48.78)
<2	21 (51.22)
First‐line treatment cycles, *n* (%^a^)	
≥3	34 (82.93)
<3	7 (17.07)
PD‐L1 expression, *n* (%^a^)	
CPS < 1	12 (29.27)
1 ≤ CPS < 5	6 (14.63)
5 ≤ CPS < 10	5 (12.20)
CPS ≥ 10	9 (21.95)
Not tested	9 (21.95)
Primary tumor site, *n* (%^a^)	
Esophagogastric junction	1 (2.40)
Non‐esophagogastric junction	40 (97.6)

Abbreviations: CPS, combined positive score; ECOG, Eastern Oncology Collaboration Group; PS, performance status; PD‐L1, programmed death‐ligand 1.

^a^ Percentages were calculated using the total number of subjects included in the SSS Population (*N*) as the denominator.

### 3.2 Efficacy

#### 3.2.1 PFS and OS

**FIGURE 1:**
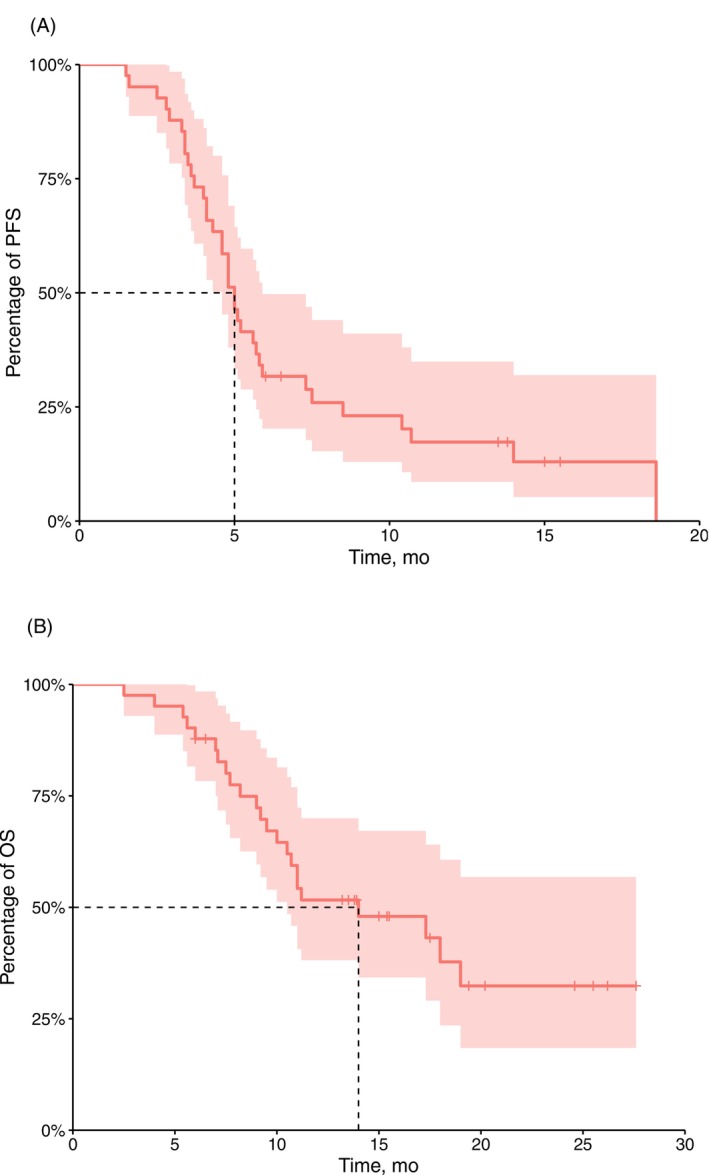
Survival curve of overall population: (A) PFS survival curve; (B) OS survival curve.

**FIGURE 2:**
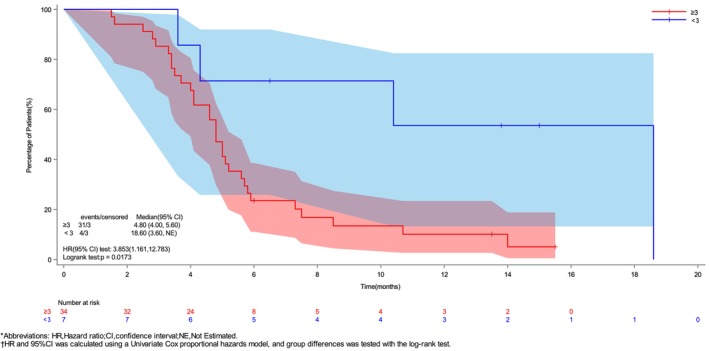
Kaplan–Meier curve of PFS by first‐line treatment cycles. The PFS of previous first‐line immunotherapy with less than three cycles is better.

As of May 1, 2025, 33 patients experienced an endpoint event. The median PFS was 5.0 months [95% confidence interval (CI), 4.1–5.8 months] (Figure 1A), and the 6 and 12‐month PFS rates were 31.7% and 17.3%, respectively. Univariate analysis indicated that age, sex, ECOG PS, Lauren classification, liver metastasis, peritoneal metastasis, and the number of metastatic organs were not independent risk factors for PFS. However, receiving ≥3 cycles of immunotherapy as a previous first‐line treatment was identified as an independent risk factor for PFS (HR 3.85, 95% CI 1.16–12.78, *p* = .02) (Supplementary Figure 2). Accordingly, patients who received <3 cycles of immunotherapy exhibited significantly better PFS compared to those who received ≥3 cycles of immunotherapy (18.6 vs. 4.8 months) (Figure 2). Subsequently, we assessed the PH assumption for all covariates before constructing the multivariable Cox model. Based on Schoenfeld residuals, the global test yielded *p* >.05 for all variables (Supplementary Table 1), confirming that the PH assumption was met. Consequently, the Cox PH model was applied. However, the multivariable analysis did not identify any statistically significant association between the covariates and the outcome event (Supplementary Table 2).

**FIGURE 3:**
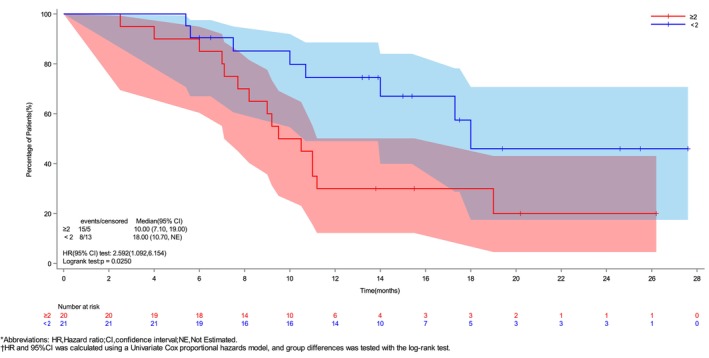
Kaplan–Meier curve of OS by number of metastatic organs. The OS of tumor metastatic organs with less than two is better. [Color figure can be viewed at wileyonlinelibrary.com]

As of May 1, 2025, 23 patients had experienced an endpoint event (death). The median follow‐up time was 12.25 months (95% CI, 10.35–14.40 months). The median OS was 14.0 months (95% CI, 10.0–NA months) (Figure 1B), and the 6‐ and 12‐month OS rates were 87.8% and 51.7%, respectively. Subgroup analysis suggested that age, sex, ECOG PS, Lauren classification, liver metastasis, peritoneal metastasis, and the number of previous first‐line immunotherapy treatments were not independent risk factors for OS. However, the number of metastatic organs was identified as an independent risk factor for OS (HR 2.59, 95% CI 1.09–6.15, *p* = .025) (Supplementary Figure 3). The patients with <2 metastatic organs had significantly better OS than patients with ≥2 metastatic organs (18 vs. 10 months) (Figure 3). Subsequently, the PH assumption was tested for all included covariates. Although the assumption was violated for some variables (Supplementary Table 3), the original model was retained without further adjustment, as the violation was not considered substantial enough to warrant modification given the current sample size and covariate structure. The multivariable analysis revealed no statistically significant associations between the covariates and the outcome event (Supplementary Table 4).

#### 3.2.2 ORR and DCR

In the efficacy analysis, 38 patients completed at least one efficacy assessment. Among them, 17, 19, and 2 patients achieved PR, SD, and PD, respectively. The ORR was 44.7% (95%CI [28.6%–61.7%]) and the DCR was 94.7% (95%CI [82.3%–99.4%]). The results of the efficacy analyses for all eligible and evaluable patients are presented in Table 2.

**TABLE 2:** Best overall response.

		*N* = 38
	*n* (%)	95% CI
Best overall response		
Complete response (CR)	0	‐
Partial response (PR)	17 (44.7)	‐
Stable disease (SD)	19 (50.0)	‐
Progressive disease (PD)	2 (5.3)	‐
Overall response rate (ORR: CR + PR)	17 (44.7)	(28.6, 61.7)
Disease control rate (DCR: CR + PR + SD)	36 (94.7)	(82.3, 99.4)

*Note*: *N*: The total number of subjects who have measurable lesions. It is the denominator for percentage (%) calculation. The exact 95% CI for the frequency distribution of each variable were computed using Clopper and Pearson method.

Abbreviations: CR, complete remission; CI, confidence interval; DCR, disease control rate; ORR, objective response rate; PD, progressive disease; PR, partial remission; SD, stable disease.

#### 3.2.3 Safety

The safety population comprised all 41 enrolled patients who received at least one dose of treatment. The frequency of treatment‐emergent AEs is summarized in Table 3. The most frequent AEs included gastrointestinal reactions, fatigue, leukopenia, neutropenia, anemia, thrombocytopenia, peripheral neuropathy, and elevated aspartate aminotransferase levels. The most commonly observed grade 3–4 AEs were neutropenia (11 patients, 26.8%), thrombocytopenia (10 patients, 24.3%), anemia (2 patients, 4.9%), febrile neutropenia (2 cases, 4.9%), and fever (1 patient, 3.2%). The overall incidence of AEs was 90.2%, while the incidence of grade 3–4 AEs was 41.5%. Notably, no treatment‐related deaths were reported.

**TABLE 3:** Summary of treatment‐related adverse events in all patients.

	All grades (*n* = 41)	≥ Grade 3 (*n* = 41)
Any treatment‐related adverse events	37 (90.2)	17 (41.5)
Platelet count decreased	25 (61.0)	10 (24.3)
Neutrophil count decreased	17 (41.5)	11 (26.8)
White blood cell count decreased	21 (51.2)	0
Anemia	20 (48.8)	2 (4.9)
Febrile neutropenia	2 (4.9)	2 (4.9)
Fever	2 (4.9)	1 (3.2)
Nausea	20 (48.8)	0
Vomiting	11 (26.8)	0
Fatigue	22 (53.7)	0
Proteinuria	6 (14.6)	0
Elevated aspartate aminotransferase	12 (29.3)	0
Elevated alanine aminotransferase	8 (19.5)	0
Decreased appetite	22 (53.7)	0
Asthenia	8 (19.5)	0
Hypoesthesia	12 (29.3)	0
Hypertension	9 (22.0)	0
Hypothyroidism	2 (4.9)	0
Hyperthyroidism	1 (3.2)	0
Amylase increased	1 (3.2)	0
Rash	4 (9.8)	0
Hand‐foot skin reaction	5 (12.2)	0
Blood thyroid stimulating hormone increased	1 (3.2)	0
Lipase increased	1 (3.2)	0

## 4 DISCUSSION

The advancement of ICI research has resulted in the establishment of first‐line treatment for GC. In contrast, the exploration of second‐line treatment options has not yielded the anticipated outcomes. The RAINBOW study and the RAINBOW‐ASIA bridging trial, conducted primarily in the Chinese population within the Asia‐Pacific region, have demonstrated that the anti‐angiogenic drug ramucirumab, in combination with taxanes, is more effective than chemotherapy alone in the second‐line treatment of advanced GC.^3^, ^10^ However, these studies were conducted prior to the advent of ICI combined with chemotherapy as a first‐line treatment for GC. Consequently, choosing an appropriate second‐line treatment regimen for advanced GC necessitates new considerations and further research evidence in the current era of immunotherapy.

Only a few studies have investigated the therapeutic efficacy of GC patients who have received prior immunotherapy. The FRUTIGA trial was a phase III randomized clinical study, which evaluated fruquintinib plus paclitaxel versus paclitaxel monotherapy as second‐line treatment for advanced GC. Although the study demonstrated a significant survival benefit for the combination therapy (median PFS 5.6 months vs. 2.7 months; HR = 0.57, *p* <.0001),^5^ notably, only 11.7% of the study population had received prior immunotherapy. A subsequent subgroup analysis indicated that the group with prior immunotherapy exhibited better efficacy than the overall population (PFS 6.4 months vs. 1.8 months, HR = 0.38, *p* = .0003).^11^ Similarly, the CheckMate649 study evaluated subsequent second‐line treatment options. The results revealed that among patients who received ramucirumab combined with taxanes as second‐line treatment, those who had previously received a PD‐1 (programmed cell death protein 1) inhibitor in combination with chemotherapy as first‐line treatment had a longer duration of first‐line therapy compared to their counterparts. This finding suggests that immunotherapy extends PFS for patients undergoing first‐line treatment and induces significant changes in the tumor immune microenvironment, potentially enhancing the effectiveness of combining anti‐angiogenic therapy with chemotherapy in the second‐line setting. Notably, subsequent treatments in CheckMate 649 were highly variable, and did not employ a standardized effective therapy. Therefore, their reported survival outcomes represent a heterogeneous treatment landscape lacking a validated later‐line standard. In contrast, our study was prospectively designed to assess a predefined, standardized protocol. Furthermore, Zhang et al. conducted a retrospective analysis of medical records from 233 consecutive patients who received antineoplastic therapy (including paclitaxel plus ramucirumab, albumin‐bound paclitaxel, or irinotecan), of whom 67 had previously received anti‐PD‐1 therapy. Among patients treated with albumin‐bound paclitaxel combined with ramucirumab (*n* = 149), the ORR was significantly higher in the anti‐PD‐1 group (60.0% vs. 20.0%, *p* <.001) along with a superior median PFS (4.8 months vs. 3.4 months, *p* = .004, HR = 0.56) compared to the non‐anti‐PD‐1 group (*p* <.001). Notably, these differences were not observed in patients receiving albumin‐bound paclitaxel monotherapy (*n* = 34) or irinotecan (*n* = 50).^12^ These results suggest that prior anti‐PD‐1 therapy may enhance tumor response to anti‐angiogenic agents combined with taxanes.

From a mechanistic perspective, the use of frontline immunotherapy may induce a “long tail” effect. This effect suggests that the therapeutic benefits for patients who respond to immunotherapy can persist over time, which could be a key mechanism enabling them to experience greater advantages from subsequent anti‐angiogenic therapies compared to their counterparts.^13^ Additionally, a synergistic effect exists between anti‐angiogenic and immunotherapeutic agents. Basic research has demonstrated that anti‐angiogenic drugs promote the overall normalization of tumor blood vessels, facilitating the delivery of therapeutic agents into the tumor, in addition to regulating the tumor microenvironment at a cellular level, thereby enhancing the anti‐tumor immune response.^14^ Furthermore, when immune cells become activated, they can secrete substantial amounts of interferon‐*γ*, which promotes the normalization of tumor vasculature. This normalized vasculature, in turn, enhances the infiltration of immune cells into the tumor, activating the immune response.^15^, ^16^ This suggests that a reciprocal enhancement exists between tumor vascular normalization and immune activation.

As a local innovative drug in China, fruquintinib has received approval from the National Medical Products Administration and the Food and Drug Administration for use as a third‐line treatment for advanced metastatic colorectal cancer, based on the findings from the FRESCO and FRESCO‐2 studies.^17^, ^18^ Fruquintinib is a highly selective VEGFR inhibitor that inhibits tumor angiogenesis, enhances the tumor microenvironment, and diminishes nutrient supply and hypoxia in tumors. Due to its low off‐target toxicity, this drug is well‐suited for use in combination therapy.^19^, ^20^ In contrast, albumin‐bound paclitaxel induces cytotoxicity by directly targeting the microtubule system of tumor cells, thereby inhibiting cell division. Thus, the combination of fruquintinib and albumin‐bound paclitaxel can synergistically inhibit tumor growth via anti‐angiogenesis and cytotoxicity.

This study explored the use of albumin‐bound paclitaxel combined with fruquintinib as second‐line therapy for patients with GC who had previously received PD‐1 inhibitor combined with chemotherapy. Notably, we found that patients who previously received <3 cycles (equivalent to two cycles) anti‐PD‐1 treatments achieved better short‐term benefits than those who received ≥3 cycles of treatment. One possible explanation for these results is that if they were inherently less sensitive to PD‐1 inhibitor (primary resistance), the resistant tumors might be more sensitive to chemotherapy or anti‐angiogenic drugs, as they are more proliferative or have a stronger dependence on angiogenesis.^21^ Chemotherapy can directly kill rapidly proliferating tumor cells, while anti‐angiogenic drugs can cut off the blood supply to tumors and inhibit their growth. Additionally, among the patients who received albumin‐bound paclitaxel combined with fruquintinib after immunotherapy exposure, those with <2 distant metastatic organs showed more significant benefits than those with ≥2 distant metastatic organs, which also provides a reference for the selection of advantageous populations in clinical treatment. However, no positive results were obtained in this study regarding the impact of liver metastasis and peritoneal metastasis on prognosis. Consequently, future studies are required to analyze larger sample sizes in this regard.

The overall efficacy and survival analysis for the entire population indicated that the combination of chemotherapy and anti‐angiogenic drugs can result in good therapeutic effects for patients with immunotherapy exposure. Previous studies have identified certain genetic characteristics associated with durable responses to ICIs. For example, high tumor mutational burden and specific genetic mutations, such as *ARID1A* abnormalities, have been linked to prolonged tumor responses. However, whether these factors can serve as reliable biomarkers to guide treatment requires further investigation in larger patient cohorts.^22^, ^23^ Furthermore, preclinical data indicate that fruquintinib combined with immunotherapy may have a synergistic anti‐tumor effect, inhibiting tumor neovascularization while promoting vascular normalization. This combination enhances the infiltration of cytotoxic T cells, reduces PD‐1‐positive CD8+ T cells, stimulates their cytotoxic function, and modulates tumor‐associated macrophages to encourage the polarization of M1 macrophages, thereby improving phagocytic activity.^24^ The patients included in this study were from an immune‐exposed population. Therefore, the potential benefits of crossover immunotherapy warrant further exploration in future prospective studies (NCT06417892).

This study has several limitations. First, the relatively small sample size may limit the statistical power and generalizability of our findings. A larger cohort would enhance the reliability of the results and allow for more robust subgroup analyses. Second, as a single‐arm prospective study, the lack of a control group makes it difficult to definitively attribute the observed effects to the intervention alone. Future randomized controlled trials with larger sample sizes are needed to validate our conclusions. Additionally, the short follow‐up duration may not fully capture long‐term outcomes or late AEs.

## 5 CONCLUSIONS

Our findings indicate that the combination of albumin‐bound paclitaxel and fruquintinib exhibits a favorable safety profile as a second‐line treatment for patients with advanced or metastatic GC, particularly in the challenging subgroup of patients who have received prior immunotherapy. These results suggest this regimen holds promise for enriching the second‐line treatment landscape. Further validation in randomized controlled trials or larger prospective studies is warranted.

## AUTHOR CONTRIBUTIONS

**Xiaoting Ma:** Data curation; formal analysis; writing – original draft. **Kai Ou:** Methodology; investigation. **Xiu Liu:** Methodology; investigation. **Biyang Cao:** Data curation; project administration. **Wenwei Yang:** Data curation; project administration. **Jingyu Lu:** Validation; formal analysis. **Letian Zhang:** Validation; formal analysis. **Qi Wang:** Investigation; formal analysis. **Lizhen Gao:** Formal analysis; investigation. **Zhichao Jiang:** Data curation; resources. **Yongkun Sun:** Data curation; resources. **Lin Yang:** Funding acquisition; writing – review and editing.

## FUNDING INFORMATION

This work was supported by the Beijing Xisike Clinical Oncology Research Foundation (Y‐HH202102‐0314), Wu Jieping Medical Foundation Scientific Research Special Funding Program (320.6750.2024‐19‐24).

## CONFLICT OF INTEREST STATEMENT

The authors declare no conflicts of interest.

## ETHICS STATEMENT

This research (ChiCTR2200059976) received ethical approval from the Institutional Review Board of the National Cancer Center/Cancer Hospital, the Chinese Academy of Medical Sciences and Peking Union Medical College. The study was conducted in compliance with the ethical principles outlined in the Declaration of Helsinki and adhered to the Good Clinical Practice guidelines established by the International Council for Harmonization. Written informed consent was obtained from all participants prior to their enrollment in the study.

## Supporting information


**Supplementary Table 1.** Proportional hazards assumption test of progression‐freesurvival.
**Supplementary Table 2**. Multivariate Cox regression analysis of progression‐free survival.
**Supplementary Table 3**. Proportional hazards assumption test of overall survival.
**Supplementary Table 4**. Multivariate Cox regression analysis of overall survival.
**Supplementary Figure 1**. CONSORT flow diagram.
**Supplementary Figure 2**. Forest plot of subgroup analysis for progression‐free.
**Supplementary Figure 3**. Forest plot of subgroup analysis for overall survival.

## Data Availability

The complete datasets generated and analyzed during this study are available in both the main manuscript and its supplementary materials. Further information is available from the corresponding author upon request.
