# Supplementary material for: Efficacy and safety of fruquintinib combined with albumin‐bound paclitaxel as second‐line therapy for advanced gastric cancer following failure of PD‐1 inhibitor‐containing treatment (TACTIC GC‐01): A phase II single‐arm study
Source: Int J Cancer. 2025 Dec 25;158(9):2219–28. doi: 10.1002/ijc.70299 (PMC12963711; doi:10.1002/ijc.70299)
Supplement: Supplementary file 1 — Supplementary Table 1. Proportional hazards assumption test of progression‐freesurvival. Supplementary Table 2. Multivariate Cox regression analysis of progression‐free survival. Supplementary Table 3. Proportional hazards assumption test of overall survival. Supplementary Table 4. Multivariate Cox regression analysis of overall survival. Supplementary Figure 1. CONSORT flow diagram. Supplementary Figure 2. Forest plot of subgroup analysis for progression‐free. Supplementary Figure 3. Forest plot of subgroup analysis for overall survival. [file IJC-158-2219-s001.pdf]

Efficacy and Safety of Fruquintinib Combined with Albumin-bound Paclitaxel as Second-line Therapy for Advanced Gastric Cancer Following Failure of PD-1 Inhibitor-containing Treatment (TACTIC GC-01): A Phase II Single-arm Study

Xiaoting Ma, Kai Ou, Xiu Liu, Biyang Cao, Wenwei Yang, Jingyu Lu, Letian Zhang, Qi Wang, Lizhen Gao, Zhichao Jiang, Yongkun Sun, Lin Yang

Page 2-5: Supplementary tables

Page 6-8: Supplementary figures

Supplementary Table 1. Proportional hazards assumption test of progression-free survival

Variable	Maximum Absolute Value	Replications	Seed	Pr>MaxAbsVal
Sex (male vs. female)	1.4662	2000	25916	0.3045
Age group (≥65 vs. <65)	2.6796	2000	25916	0.1405
Lauren type (Diffuse type vs. Intestinal type)	1.1564	2000	25916	0.7210
Lauren type (Mixed type vs. Intestinal type)	1.5385	2000	25916	0.2360
Liver metastasis (YES vs. NO)	2.0811	2000	25916	0.3975
Peritoneal Metastasis (YES vs. NO)	2.5242	2000	25916	0.4415
First-line Treatment Cycles (≥3 vs. <3)	0.7672	2000	25916	0.5525
Number of Metastatic Organs (≥2 vs. <2)	2.6012	2000	25916	0.0990
PD-L1 expression (1≤CPS < 5 vs. CPS < 1)	1.5579	2000	25916	0.5150
PD-L1 expression (5≤CPS < 10 vs. CPS < 1)	1.3773	2000	25916	0.1590
PD-L1 expression (CPS≥10 vs. CPS < 1)	2.7706	2000	25916	0.0780
ECOG PS(1 vs. 0)	2.7470	2000	25916	0.0920

*Supremum test was performed to test proportional hazards assumption.P>0.05, supporting the assumption

Supplementary Table 2. Multivariate Cox Regression Analysis of Progression-free Survival

Variable	Coefficient	Standard Error	HR(95%CI)	P-value
Sex (male vs. female)	1.781	1.151	5.935(0.621,56.679)	0.1219
Age group (≥65 vs. <65)	-0.091	1.697	0.913(0.033,25.415)	0.9572
Lauren type (Diffuse type vs. Intestinal type)	2.691	1.703	14.744(0.524,415.234)	0.1141
Lauren type (Mixed type vs. Intestinal type)	1.707	1.200	5.510(0.525,57.881)	0.1550
Liver metastasis (YES vs. NO)	2.731	2.332	15.343(0.159,1482.204)	0.2416
Peritoneal Metastasis (YES vs. NO)	4.570	3.031	96.579(0.254,36721.112)	0.1316
First-line Treatment Cycles (≥3 vs. <3)	1.443	1.186	4.234(0.415,43.240)	0.2235
Number of Metastatic Organs (≥2 vs. <2)	-2.583	1.878	0.076(0.002,2.997)	0.1690
PD-L1 expression (1≤CPS < 5 vs. CPS < 1)	1.750	2.491	5.756(0.044,759.681)	0.4823
PD-L1 expression (5≤CPS < 10 vs. CPS < 1)	-1.384	2.075	0.251(0.004,14.625)	0.5047
PD-L1 expression (CPS≥10 vs. CPS < 1)	0.527	1.677	1.694(0.063,45.302)	0.7533
ECOG PS(1 vs. 0)	0.544	1.362	1.723(0.119,24.866)	0.6896

*Lauren type (3 categories) was derived into 2 dummy variables, with

Intestinal type' as the reference,and 'not tested' responses were treated as missing and excluded from multivariate models.'

†PD-L1 expression (4 categories) was derived into 3 dummy variables, with ' CPS <

1' as the reference,and 'not tested' responses were treated as missing and excluded from multivariate models.

Supplementary Table 3. Proportional hazards assumption test of overall survival

Variable	Coefficient	Standard Error	HR(95%CI)	P-value
Sex (male vs. female)	2.0215	2000	25916	0.2875
Age group (≥65 vs. <65)	1.5064	2000	25916	0.3280
Lauren type (Diffuse type vs. Intestinal type)	1.8402	2000	25916	0.2780
Lauren type (Mixed type vs. Intestinal type)	1.7961	2000	25916	0.1505
Liver metastasis (YES vs. NO)	2004.9014	2000	25916	0.5260
Peritoneal Metastasis (YES vs. NO)	3751.2034	2000	25916	0.3375
First-line Treatment Cycles (≥3 vs. <3)	1.0281	2000	25916	0.2275
Number of Metastatic Organs (≥2 vs. <2)	4370.4431	2000	25916	0.2455
PD-L1 expression (1≤CPS＜5 vs. CPS＜1)	2.3941	2000	25916	0.2030
PD-L1 expression (5≤CPS < 10 vs. CPS < 1)	0.0002	2000	25916	0.0060
PD-L1 expression (CPS≥10 vs. CPS < 1)	2.8869	2000	25916	0.2385
ECOG PS(1 vs. 0)	0.7715	2000	25916	0.2805

***Supremum test was performed to test proportional hazards assumption.P>0.05, supporting the assumption.**

†Given the small sample size and a relatively large number of covariates, it appears unnecessary to conduct further handling for individual variables that do not satisfy the PH assumption, such as stratification.

Supplementary Table 4. Multivariate Cox Regression Analysis of Overall Survival

Variable	Coefficient	Standard Error	HR(95%CI)	P-value
Sex (male vs. female)	3.272	2.099	26.374(0.431,1615.364)	0.1191
Age group (≥65 vs. <65)	4.490	2.568	89.082(0.580,13672.997)	0.0804
Lauren type (Diffuse type vs. Intestinal type)	-0.204	2.087	0.816(0.014,48.758)	0.9222
Lauren type (Mixed type vs. Intestinal type)	-4.403	2.097	0.012(0.000,0.746)	0.0358
Liver metastasis (YES vs. NO)	-20.799	5308.004	0.000(0.000,NE)	0.9969
Peritoneal Metastasis (YES vs. NO)	-10.627	5308.005	0.000(0.000,NE)	0.9984
First-line Treatment Cycles (≥3 vs. <3)	-1.694	1.544	0.184(0.009,3.790)	0.2726
Number of Metastatic Organs (≥2 vs. <2)	17.803	5308.004	5.394E+07(0.000,NE)	0.9973
PD-L1 expression (1≤CPS < 5 vs. CPS < 1)	6.811	3.795	908.009(0.534,1544063.468)	0.0727
PD-L1 expression (5≤CPS < 10 vs. CPS < 1)	-13.979	5039.015	0.000(0.000,NE)	0.9978
PD-L1 expression (CPS≥10 vs. CPS < 1)	4.806	2.748	122.210(0.560,26663.388)	0.0803
ECOG PS(1 vs. 0)	-0.635	1.529	0.530(0.026,10.617)	0.6782

*Lauren type (3 categories) was derived into 2 dummy variables, with

Intestinal type' as the reference,and 'not tested' responses were treated as missing and excluded from multivariate models.'

†PD-L1 expression (4 categories) was derived into 3 dummy variables, with ' CPS <

1' as the reference,and 'not tested' responses were treated as missing and excluded from multivariate models.

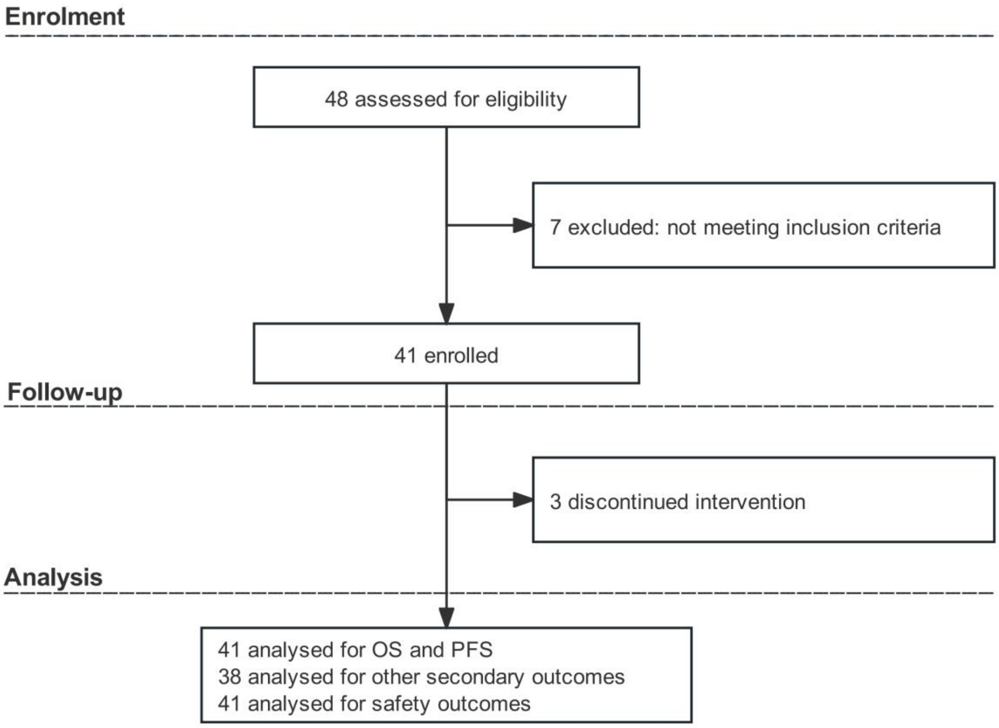

Supplementary Figure 1. *CONSORT Flow Diagram.* A total of 48 patients were screened, and 41 were finally enrolled. None of the patients dropped out of the study midway. All 41 participants underwent safety assessment, and 38 of them underwent efficacy assessment.

Subgroup	events/total(%)	Median(95%CI)	HR (95% CI)	P value
Overall	35/41(85.4)	5.00 (4.10, 5.80)		
Sex				
Male	23/27(85.2)	5.00 (4.10, 7.50)	0.804 (0.396, 1.632)	0.5452
Female(reference)	12/14(85.7)	4.90 (3.40, 5.90)		
Age groups				
≥65	10/11(90.9)	5.80 (4.30, 10.70)	0.787 (0.375, 1.653)	0.5271
< 65(reference)	25/30(83.3)	4.70 (3.60, 5.60)		
Lauren type				
Intestinal type(reference)	8/10(80.0)	6.15 (2.80, NE)		
Diffuse type	7/9(77.8)	4.00 (2.90, 10.40)	1.616 (0.561, 4.653)	0.3737
Mixed type	5/5(100.0)	5.80 (1.50, NE)	2.116 (0.649, 6.906)	0.2141
Liver metastasis				
YES	11/13(84.6)	4.80 (3.40, 5.70)	1.169 (0.567, 2.411)	0.6732
NO(reference)	24/28(85.7)	5.00 (4.10, 7.30)		
Peritoneal Metastasis				
YES	11/12(91.7)	4.80 (3.40, 7.30)	1.277 (0.617, 2.642)	0.5105
NO(reference)	24/29(82.8)	5.00 (4.00, 7.50)		
Number of Metastatic Organs				
≥2	18/20(90.0)	4.60 (3.50, 5.20)	1.527 (0.772, 3.022)	0.2240
< 2(reference)	17/21(81.0)	5.70 (4.30, 8.50)		
First-line Treatment Cycles				
≥3	31/34(91.2)	4.80 (4.00, 5.60)	3.853 (1.161, 12.783)	0.0275
< 3(reference)	4/7(57.1)	18.60 (3.60, NE)		
PD-L1 expression				
CPS < 1(reference)	12/12(100.0)	4.80 (3.60, 5.60)		
1≤CPS < 5	5/6(83.3)	4.15 (2.80, NE)	0.859 (0.295, 2.496)	0.7794
5≤CPS < 10	4/5(80.0)	5.00 (1.60, NE)	0.741 (0.234, 2.342)	0.6094
CPS≥10	6/9(66.7)	5.80 (1.50, NE)	0.557 (0.205, 1.517)	0.2522
ECOG PS				
0(reference)	18/23(78.3)	4.80 (3.50, 5.80)		
1	17/18(94.4)	5.05 (4.10, 8.50)	0.973 (0.496, 1.911)	0.9376

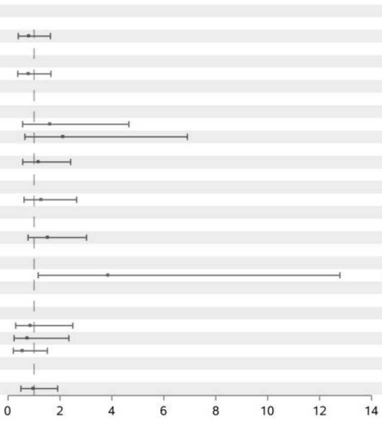

*Hazard ratios(HRs) with 95% confidence intervals (CIs) and P values were calculated using a Univariate Cox proportional hazards model.

Supplementary Figure 2. *Forest Plot of Subgroup Analysis for Progression-Free Survival.* Log-Rank test indicated that the number of previous first-line immunotherapy was an independent risk factor for PFS.

Subgroup	events/total(%)	Median(95%CI)	HR (95% CI)	P value
Overall	23/41(56.1)	14.00 (10.00, NE)		
Sex				
Male	15/27(55.6)	14.00 (10.00, NE)	0.837 (0.354, 1.979)	0.6855
Female(reference)	8/14(57.1)	11.20 (5.60, NE)		
Age groups				
≥65	6/11(54.5)	14.00 (7.10, NE)	0.919 (0.362, 2.333)	0.8589
< 65(reference)	17/30(56.7)	11.20 (9.00, NE)		
Lauren type				
Intestinal type(reference)	4/10(40.0)	NE (7.00, NE)		
Diffuse type	5/9(55.6)	17.30 (4.00, NE)	1.714 (0.459, 6.404)	0.4232
Mixed type	2/5(40.0)	NE (9.00, NE)	1.030 (0.188, 5.646)	0.9724
Liver metastasis				
YES	7/13(53.8)	11.00 (7.10, NE)	0.914 (0.375, 2.226)	0.8424
NO(reference)	16/28(57.1)	17.30 (8.20, 19.00)		
Peritoneal Metastasis				
YES	8/12(66.7)	10.00 (7.00, NE)	1.803 (0.736, 4.418)	0.1971
NO(reference)	15/29(51.7)	17.30 (10.70, NE)		
Number of Metastatic Organs				
≥2	15/20(75.0)	10.00 (7.10, 19.00)	2.592 (1.092, 6.154)	0.0309
< 2(reference)	8/21(38.1)	18.00 (10.70, NE)		
First-line Treatment Cycles				
≥3	21/34(61.8)	11.20 (9.50, 19.00)	2.172 (0.509, 9.272)	0.2950
< 3(reference)	2/7(28.6)	NE (7.50, NE)		
PD-L1 expression				
CPS < 1(reference)	9/12(75.0)	10.00 (7.00, NE)		
1≤CPS < 5	4/6(66.7)	17.30 (7.70, NE)	0.527 (0.158, 1.752)	0.2960
5≤CPS < 10	2/5(40.0)	18.00 (6.00, NE)	0.343 (0.073, 1.619)	0.1768
CPS≥10	3/9(33.3)	NE (5.40, NE)	0.361 (0.097, 1.350)	0.1301
ECOG PS				
0(reference)	15/23(65.2)	10.70 (8.20, 18.00)		
1	8/18(44.4)	19.00 (9.50, NE)	0.427 (0.175, 1.040)	0.0609

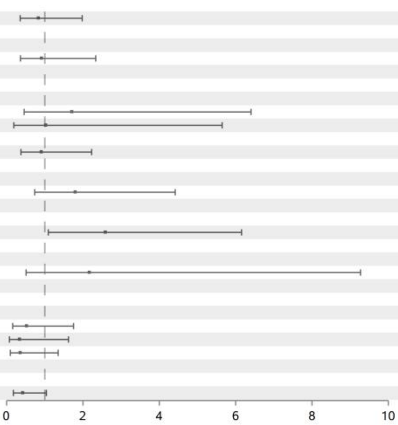

*Hazard ratios(HRs) with 95% confidence intervals (CIs) and P values were calculated using a Univariate Cox proportional hazards model.

Supplementary Figure 3. *Forest Plot of Subgroup Analysis for Overall Survival.* Log-Rank test indicated that the number of metastatic organs was an independent risk factor for OS.
